# Enhanced Physical Capacity and Gastrointestinal Symptom Improvement in Southern Italian IBS Patients following Three Months of Moderate Aerobic Exercise

**DOI:** 10.3390/jcm12216786

**Published:** 2023-10-26

**Authors:** Antonella Bianco, Francesco Russo, Isabella Franco, Giuseppe Riezzo, Rossella Donghia, Ritanna Curci, Caterina Bonfiglio, Laura Prospero, Benedetta D’Attoma, Antonia Ignazzi, Angelo Campanella, Alberto Ruben Osella

**Affiliations:** 1 Laboratory of Epidemiology and Statistics, National Institute of Gastroenterology IRCCS “Saverio de Bellis”, 70013 Castellana Grotte, Italy; antonella.bianco@irccsdebellis.it (A.B.); isabella.franco@irccsdebellis.it (I.F.); ritanna.curci@irccsdebellis.it (R.C.); catia.bonfiglio@irccsdebellis.it (C.B.); angelo.campanella@irccsdebellis.it (A.C.); arosella@irccsdebellis.it (A.R.O.); 2 Functional Gastrointestinal Disorders Research Group, National Institute of Gastroenterology IRCCS “Saverio de Bellis”, 70013 Castellana Grotte, Italy; giuseppe.riezzo@irccsdebellis.it (G.R.); laura.prospero@irccsdebellis.it (L.P.); benedetta.dattoma@irccsdebellis.it (B.D.); antonia.ignazzi@irccsdebellis.it (A.I.); 3 Data Science Unit, National Institute of Gastroenterology IRCCS “Saverio de Bellis”, 70013 Castellana Grotte, Italy; rossella.donghia@irccsdebellis.it

**Keywords:** aerobic exercise, irritable bowel syndrome, Global Physical Capacity Score, physical capacity

## Abstract

Moderate-intensity aerobic exercise improves gastrointestinal (GI) health and alleviates irritable bowel syndrome (IBS) symptoms. This study explored its effects on physical capacity (PC) and IBS symptoms in 40 patients from Southern Italy (11 males, 29 females; 52.10 ± 7.72 years). The exercise program involved moderate-intensity aerobic exercise (60/75% of HRmax) for at least 180 min per week. Before and after the intervention, participants completed the IBS-SSS questionnaire to assess IBS symptoms, reported their physical activity levels, and underwent field tests to evaluate PC. PC was quantified as the Global Physical Capacity Score (GPCS). A total of 38 subjects (21 males, 17 females; 53.71 ± 7.27 years) without lower GI symptoms served as a No IBS group. No significant differences were found between IBS patients and No IBS subjects, except for the symptom score, as expected. After the exercise, all participants experienced significant improvements in both IBS symptoms and PC. Higher PC levels correlated with greater benefits in IBS symptomatology, especially with GPCS reaching above-average values. Engaging in moderate-intensity aerobic exercise for at least 180 min per week positively impacts IBS symptoms and PC. Monitoring GPCS in IBS patients provides insights into the connection between physical activity and symptom severity, aiding healthcare professionals in tailoring effective treatment plans.

## 1. Introduction

Irritable bowel syndrome (IBS) is a prevalent functional gastrointestinal (GI) disorder, affecting approximately 9–22% of the European adult population [1,2]. This syndrome is characterized by abdominal pain or discomfort linked to altered bowel habits without an identifiable organic cause [3]. In addition to pharmacological treatments, there has been growing interest in alternative approaches to manage this condition better. Some of the most effective alternative treatments for IBS include acupuncture, herbal remedies, and mind–body techniques [4]. Peppermint oil, ginger, and aloe have been widely used as herbal remedies for treating IBS [5]. Psyllium powder and L-Glutamine have also been considered alternative natural remedies that may help with IBS [6]. Acupuncture has proven effective for treating chronic pain, but the studies are mixed on whether this treatment works for IBS [7].

Food is often considered a triggering factor for IBS symptoms, and diet is crucial to preventing and managing this functional disorder. However, there is no one-size-fits-all diet for individuals with IBS. Nonetheless, several dietary changes can help alleviate symptoms. Avoiding caffeine, alcohol, fatty foods, and spicy foods can also be helpful for some IBS patients [8]. Several studies have shown that a low FODMAPs (fermentable oligo-, di-, monosaccharides, and polyols) diet leads to a clinical response in 50–80% of IBS patients, particularly improving bloating, flatulence, and diarrhea [9]. In this context, our research group recently published articles highlighting the benefits of a low FODMAP diet [10] and a diet based on products derived from a new cereal called tritordeum [11]. Both dietary approaches have significantly improved GI symptoms.

As widely documented in the literature, physical activity (PA) plays a key role in the prevention and treatment of various diseases [12,13,14,15], can improve general health, and reduce stress levels. In addition, it exerts numerous positive and protective effects on the GI tract [16,17], such as relieving constipation [18] and improving digestive processes by increasing the speed of stool movement. Furthermore, Villoria et al. [19] demonstrated that PA reduces symptoms such as abdominal bloating by increasing intestinal gas clearance. The same group [20] previously demonstrated that PA improved gas transit and abdominal distension in healthy subjects but not the perception of bloating. A long-term follow-up study [21] showed that a placebo effect alone cannot explain PA’s effects. A pure placebo effect would have to be decreased during follow-up.

From this perspective, PA can be a valuable tool for managing IBS, as increased participation in moderate PA has been associated with improved IBS symptoms. Several studies on IBS recommend an average prescription of 30 to 60 min of moderate-intensity aerobic PA, 3 to 5 times per week, for a minimum of 12 weeks [17]. Walking is the most common form of exercise, often complementary to other physical exercises [22]. It is known that regular walking, independent of other types of physical exercise, can improve risk factors for cardiovascular diseases, including diastolic blood pressure and lipid profiles, and reduces the risk of general mortality [23,24,25] and type 2 diabetes [26], and brings additional benefits in that it improves self-esteem, alleviates symptoms of depression and anxiety, and improves mood [27,28]. The advantages of using walking as a therapeutic tool are numerous: it can be performed at different speeds (and thus intensities), easily monitored by heart rate monitors, in groups or alone, and without special equipment or clothing.

In this framework, the walking group is a potentially interesting PA intervention as the dynamics and social cohesion of walking groups can have supportive effects that encourage and sustain adherence and positive attitudes towards PA [29], companionship, and the shared experience of well-being [30].

Regular walking, as well as regular PA, has many health effects related to physical capacity (PC) and can be assessed using various field tests. Unlike PA, which is related to the movements that people perform, PC is a set of attributes that people have or can achieve. The components of PC related to health [31] are: (a) cardiorespiratory capacity, (b) muscular resistance endurance, (c) muscle strength, (d) body composition, and (e) flexibility.

PC has been identified as an indicator of general health [32] and could also be a possible predictor of the response to PA treatment in IBS patients.

Based on the published evidence, we hypothesized that a programmed and controlled PA intervention could lead to an overall benefit in patients with IBS. In this framework, the study aimed to estimate the effect of a standardized PA program on the intensity and frequency of GI symptoms and the PC of IBS patients in Southern Italy.

## 2. Materials and Methods

### 2.1. Participants

Subjects were recruited in the study by the Functional Gastrointestinal Disorders Research Group in collaboration with the Laboratory of Epidemiology and Statistics of the National Institute of Gastroenterology IRCCS “Saverio de Bellis” Castellana Grotte, Italy. The project started in May 2022 and is still ongoing. The trial was registered at www.clinicaltrial.gov (accessed on 08 July 2022) (registration number NCT05453084).

### 2.2. Study Design

This study included adults attending the Outpatient Clinic for Celiac Disease and Functional Disorders who met the Rome III-IV criteria for IBS or were referred by local General Practitioners. The inclusion criteria were: (1) age 18–65 years, (2) availability to participate in the walking group, (3) in possession of a medical certificate of non-competitive sports fitness; and the exclusion criteria comprised: (1) presence of serious cardiac, hepatic, neurological or psychiatric diseases, (2) gastrointestinal disorders other than IBS, (3) subjects who have previously followed a low FODMAPs, vegan or gluten-free diet, (4) patients using antidepressants (5) significant orthopedic or neuromuscular limitations, (6) absolute contraindications to exercise. Furthermore, subjects who missed more than 20% of their training sessions would have been excluded from the analysis.

The study was conducted following the Helsinki Declaration and approved by the local Ethics Committee (Prot. N. 167/CE De Bellis).

### 2.3. Data Collection

**Figure 1:**
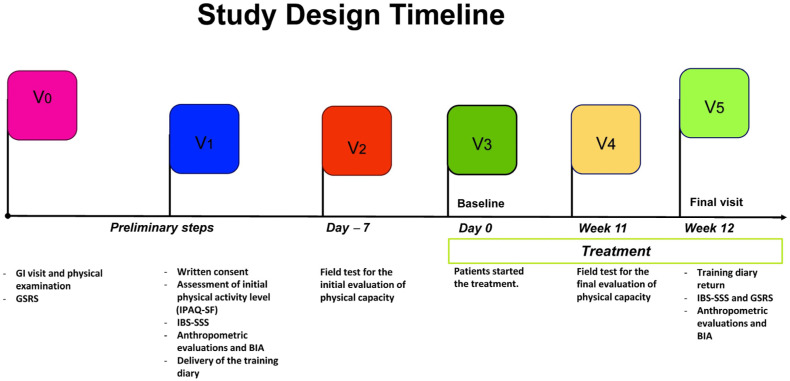
Study Design Timeline; GI: gastrointestinal; GSRS: Gastrointestinal Symptom Rating Scale; IBS-SSS: IBS-Severity Scoring System; BIA: bioelectrical impedance analysis.

During enrollment, participants signed informed consent and completed a structured questionnaire collecting data about sociodemographic aspects, medical history, and lifestyle. PA information was collected using the validated International Physical Activity Questionnaire, Short Form (IPAQ-SF) [33]. Patients also completed the symptom questionnaires Gastrointestinal Symptom Rating Scale (GSRS) for screening patients with IBS. The IBS-Severity Scoring System—IBS-SSS was administered to evaluate the selected patients’ symptom intensity and frequency scores. Trained staff collected fast blood samples for biochemical assessments, anthropometric measurements (weight, height, waist circumference), and bio-impedance analysis. All measurements were taken at the start of the project and after 90 days. The timeline of the study design is shown in Figure 1.

### 2.4. Anthropometric and Bioelectrical Impedance Analysis (BIA) Parameters

The study assessed various anthropometric parameters to investigate the subjects’ physical characteristics. The parameters included height, weight, body mass index (BMI), mid-upper arm, waist, and hip circumferences. Accurate measurements were obtained using a SECA 700 mechanical column scale and a SECA 220 altimeter (INTERMED S.r.l., Milan, Italy) for weight and height assessment, facilitating the subsequent calculation of BMI (kg/m^2^).

To ensure uniformity, a stringent protocol was followed for individuals undergoing bioelectrical impedance analysis (BIA). All participants observed a minimum 4 h fasting period and refrained from alcohol consumption and strenuous exercise in the preceding 12 h.

The BIA procedure involved the injection of a continuous sinusoidal current (800 A) with a frequency of 50 kHz. The BIA 101 BIVA PRO instrument (Akern SRL, Pontassieve, Italy) was utilized for all measurements, aligning with the rigorous standards recommended by the European Society for Parenteral and Enteral Nutrition [34].

Parameters such as resistance (Rz) and reactance (Xc) of human tissue were measured through BIA. The same instrument was used to assess body cell mass, fat-free mass, fat mass, total body water, and extracellular water. Specialized software (Bodygram PLUS Software v. 1.0, Akern SRL, Pontassieve, Italy) facilitated the calculation of these parameters based on the obtained Rz and Xc values. The phase angle, derived as the arctangent of the Xc/Rz ratio, was also computed as a crucial metric in the evaluation.

### 2.5. Training Diary

All participants filled out their daily diary, indicating the type of PA performed and the duration. At the end of each day, they reported the total number of daily steps monitored by their heart rate monitor. The diary was used to motivate patients to be physically active, compare participation in the walking group, and know and quantify the PA performed in addition to the proposed work.

When the training diary was handed over to the participants, they were given all the information on how to fill it in correctly.

### 2.6. Exercise Protocol

#### 2.6.1. Physical Capacity Assessment Tests

Three field tests were performed to assess the subjects’ basic conditions and establish the most appropriate intensity of the training program. These included cardiorespiratory capacity, evaluated with the 2 km walk test [35], and strength and flexibility, assessed with the Hand Grip and Sit and Reach tests [36,37]. Subjects performed these tests at the beginning of the project and the end of the three months.

The week before the field tests (3 sessions of 60 min), the participants were adequately instructed on how to carry out the tests correctly so that the results would be as reliable as possible. In these 3 preliminary sessions, the experts explained the correct walking technique and corrected any problems, sensitized the participants on using suitable technical shoes to avoid injuries, and ensured each heart rate monitor functioned correctly and answered any questions.

As far as possible, the tests were reproduced under the same conditions: (a) in the same place, (b) supervised by the same operators, (c) at the same time, and (d) monitored with the same instruments.

#### 2.6.2. Exercise Intervention

PA, organized in “Walking Groups”, was structured as follows:

- Frequency. The aerobic exercise was performed outdoors on an urban route thrice a week, on non-consecutive days, for 12 weeks.
- Intensity. The aerobic exercise intensity was moderate (60/75% of HR max); it was monitored through the heart rate monitor and was personalized through Tanaka’s formula [38]. In addition, the Talk Test [39] and the Borg scale [40] were used to measure the rhythm and the perception of fatigue, respectively.
- Type. The aerobic exercise type was walking, ranging from 5 to 10 km/h.
- Time. Each walk had a duration of 60′ for a total of 180′ per week; the single outing lasting 60′ was structured as follows: Warm-up: 5′; Normal walk: 10′; Sustained walking: 30′; Fast walking: 10′; Cool-down: 5′. The entire activity was supervised by experts (Graduates in Preventive and Adapted Physical Activity Science and Techniques), and the presence of the participants at each training session was strictly registered.

#### 2.6.3. Exposure—Global Physical Capacity Score

PC was measured by a series of motor tests of varying difficulty, validated in adult subjects, to assess cardiorespiratory capacity, strength, and flexibility. A PC score was then calculated using the results of each test. Each physical test was scored from 0 to 2 using performance categories (e.g., performance above average = 2 points, average = 1 point, below average or unable to complete the test = 0 points). Then, the scores of the 3 tests were added to obtain an overall physical ability score (possible range of scores between 0 and 6 points). The GPCS used in the present study was adapted from the approach previously proposed by Bouchard et al. [41]. An advantage of calculating and using GPCS is that it provides an overall measure of physical performance that considers several tasks related to daily activities, unlike each test taken individually.

### 2.7. Outcome Assessment—IBS Severity Scoring System (IBS-SSS)

To assess the GI symptom profile, the GSRS and the IBS-SSS questionnaires were administered. The first is The GSRS is a disease-specific instrument of 15 items combined into five symptom clusters depicting “Reflux”, “Abdominal pain”, “Indigestion”, “Diarrhea”, and “Constipation”. The GSRS has a seven-point Likert-type scale where 1 represents the absence of troublesome symptoms, and 7 represents very troubling symptoms. The latter is a validated questionnaire that consists of five items with a score ranging from 0 to 500: “Abdominal pain intensity”, “Abdominal pain frequency”, “Abdominal distension”, “Dissatisfaction with bowel habit”, and “Interference on life in general”. The applied cut-off score to determine the IBS severity was as follows; >75–175 for “mild IBS”, 175–300 for “moderate IBS”, and >300 for “severe IBS” [42].

### 2.8. Statistical Analysis

Patients’ characteristics were described by mean ± SD (or median values where necessary) and frequency (%) for continuous and categorical variables. For continuous data, the Kruskal–Wallis with Dunn’s post-test was used to assess differences among the groups (No IBS subjects, IBS patients pre- and after treatment). The Wilcoxon test was performed to evaluate the effects of treatment on the single variables in IBS patients. Chi-square tests were used to compare categorical data (e.g., results from the 2Km Walking Test, Sit and Reach Test, Hand-Grip Test, and IPAQ). The main outcome is by an ordered logistic regression. As there were two repeated measurements of the outcome, we performed a mixed ordinal logistic model to account for the study’s design and the data’s correlation structure. After fitting the ordinal logistic model, we obtained predictions (probabilities) using post-estimation tools. The statistical analysis was performed with Stata Statistical Software 18 (Corp, 4905 Lakeway Drive, College Station, TX, USA). The analyses were conducted using RStudio (“Prairie Trillium” Release) for the graphics.

## 3. Results

**Figure 2:**
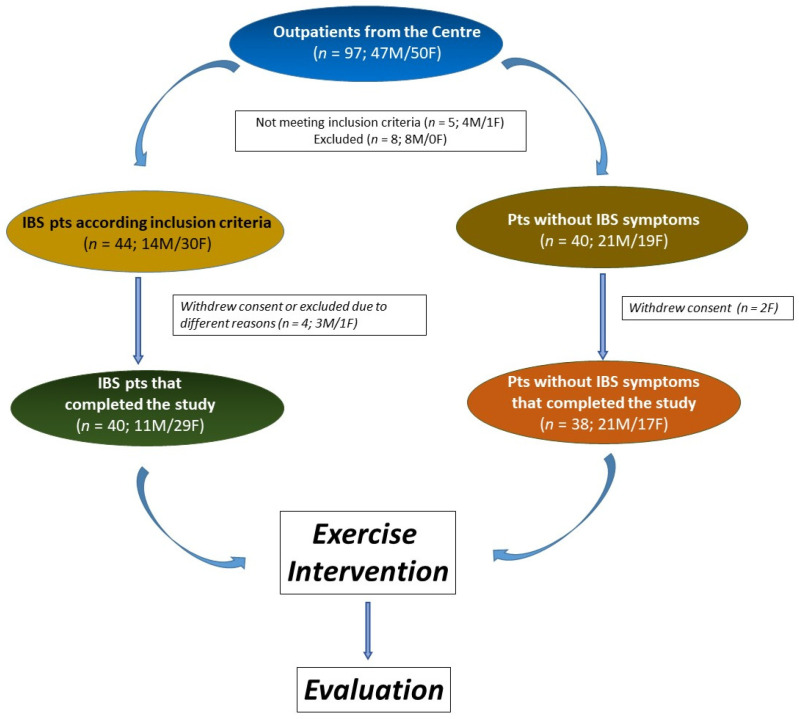
The flowchart of the study.

The study involved 78 participants, with 40 identified as IBS patients. The No IBS group comprised 38 individuals without lower gut symptoms but experiencing mild symptoms of upper gut diseases, such as dyspepsia or gastroesophageal reflux. The study flow is depicted in Figure 2. All participants adhered to the same exercise program.

### 3.1. Patient Characteristics

The participants’ characteristics are shown in Table 1. Comparing the three groups, there were no significant differences regarding anthropometric parameters expressed as BMI, waist and hip circumferences, and waist/hip ratio. As for BIA parameters, the No IBS subjects had slightly but significantly, higher mean values of BCM, FFM, and TBW than IBS subjects, indicating better health and nutritional conditions and hydration status. Furthermore, these parameters remained unchanged at the end of the exercise program in subjects with IBS. Conversely, the GPCS after the PA program was significantly higher (*p* < 0.05) than No IBS subjects and IBS patients before treatment.

The results from the IBS-SSS questionnaire indicated a clear difference between IBS patients and No IBS subjects at baseline, as expected. Considering the IBS-SSS items after the programmed exercise, a marked and significant reduction (*p* < 0.01) in single and total items was found. Moreover, the mean total IBS-SSS score changed from moderate to mild after the programmed exercise. The temporal trend of GPCS and IBS-SSS scores and some anthropometrical data (GPCS and IBS-SSS scores, BMI, waist circumference, and hip circumference) during intervention time are reported in Appendix A.

Concerning the scores of the 2Km Walking Test, Sit and Reach Test, Hand-Grip Test, and IPAQ, there were no significant differences among the groups.

### 3.2. Modifications in IBS Categories Based on IBS-SSS and GPCS

**Figure 3:**
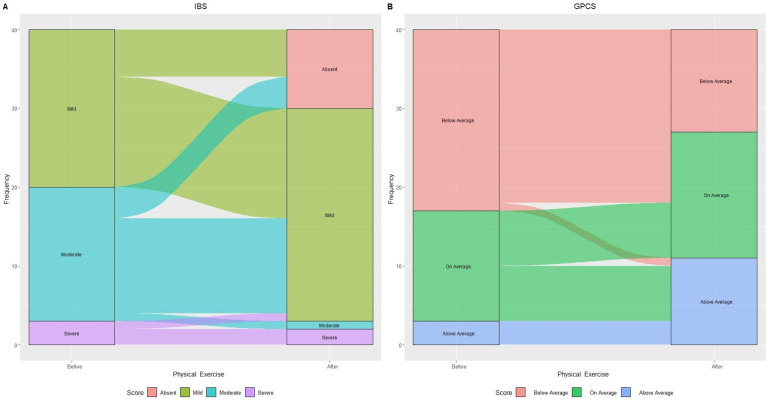
Alluvial plot showing patient flow in relation to IBS categories: “Mild”, “Moderate”, and “Severe” (**A**) and Global Physical Capacity score (GPCS): “Below Average”, “On average”, and “Above Average” (**B**) before and after physical exercise.

Figure 3 illustrates changes in IBS categories based on IBS-SSS score and GPCS after 90 days of treatment.

The aerobic exercise led to 30% (n = 6) of initially “Mild IBS” and 23.5% (n = 4) of “Moderate IBS” subjects transitioning to “Absent IBS symptoms”.

Within “Moderate IBS”, all but one (70.5% or n = 12) experienced symptom reduction, now classified as “Mild IBS.” One person with “Severe IBS” improved to “Mild IBS”, resulting in a 60% increase in that category. GPCS showed a 43.5% decrease in “Below average” subjects and doubled the “Above average” number, increasing from 3 to 11 individuals.

The multivariable ordered logistic mixed model (Table 2), which included age (gender, BMI, waist and hip circumference, and IBS severity categories), showed the odds ratio for the highest GPCS category (above the mean) to be 0.04. Subjects above the mean GPCS at the end of the intervention (score of 5 to 6) significantly reduced IBS symptoms and were less likely to develop the same symptoms. Furthermore, the higher the fitness score, the more protective effect of the intervention was on IBS symptoms.

## 4. Discussion

Present findings show that increasing PA and improving PC could reduce the IBS symptoms, highlighting the positive effects of a structured and controlled aerobic activity intervention lasting 90 days, three times a week (180 min). As expected, 3 months of PA significantly affected the IBS symptom profile. As reported by the IBS-SSS, the total score significantly reduced by 39% compared to baseline, and the same occurred for the Abdominal Distension. In addition, we observed that subjects with an “above average” value in the GPCS had a more significant effect in reducing IBS symptoms than the other categories of the GPCS (namely, “below average” and “average”).

There is well-established evidence supporting the health benefits of PA, making it a common recommendation for health promotion and prevention [43]. Regular participation in PA reduces the risk of premature mortality and the development of over 25 chronic medical conditions [43]. Most international PA guidelines for healthy individuals and clinical populations recommend a minimum of 150 min per week of moderate to vigorous intensity PA (MVPA) [44]. Studies have reported that individuals meeting or exceeding international recommendations experienced a 20–30% reduction in the risk of premature mortality and chronic diseases [43].

In this framework, greater risk reductions were observed when objective measurements of health-related PC were utilized [43]. Monitoring PC through validated and accurate tests provides reliable information about the proposed exercise intervention, the improvements achieved by each individual, and the possibility of modifying the program if necessary. Our study utilized a summary score of different field tests (GPCS) to investigate the hypothesized association between improved PC and reduced IBS symptoms. Present findings demonstrated that improved IBS symptoms were effectively accompanied by increased GPCS, which resulted from higher cardiorespiratory capacity, muscle mass, strength, endurance, and flexibility through the exercise program.

Currently, limited and conflicting data are available regarding the association between PA and IBS. While there is evidence of health benefits from moderate exercise in patients with inflammatory bowel disease or functional GI disorders, the safety of more intense exercise has not been clearly established [45].

Controlled and moderate PA has been consistently linked to many health benefits, including improving gastrointestinal (GI) symptoms. While the precise physiological mechanisms remain not entirely elucidated, several factors contribute to this positive relationship.

One significant contributor is the enhancement of gut motility achieved through increased bowel contractions and reduced transit time [46]. This heightened motility can positively impact digestion and overall GI function. Additionally, controlled PA fosters improved blood flow, promoting the GI tract’s health. The positive effects extend to the modulation of inflammation through anti-inflammatory mechanisms, further contributing to GI well-being [46].

PA plays a role in stress reduction through cortisol regulation, which has implications for GI health, given the well-established link between stress and gastrointestinal symptoms. Moreover, there is evidence of positive effects on gut microbiota diversity, with regular exercise potentially influencing the composition and function of the microbial community in the digestive system [47].

Hormonal regulation is another key aspect, with controlled PA potentially leading to the release of glucagon-like peptide-1 (GLP-1), which is associated with improved gut function. Weight management is also crucial, as PA can aid in preventing obesity-related GI issues [48].

Furthermore, the positive impact of moderate PA extends to enhanced immune function, which plays a vital role in overall GI health [49]. Finally, the mind–body connection is a holistic aspect that influences psychological well-being, positively impacting GI health overall [50].

It is important to note that individual responses to PA may vary significantly among individuals, highlighting the need for personalized approaches to understanding and harnessing the GI benefits of controlled and moderate PA.

Indeed, some previous studies have reported that moderate PA improves IBS symptoms. On the other hand, a systematic review evidenced that increasing exercise intensity and duration can paradoxically lead to intestinal damage, increased permeability, endotoxemia, impaired gastric emptying, slowed small intestinal transit, and malabsorption. Significant GI disturbances occur with exercise stress lasting ≥2 h at 60% VO2 max, regardless of the fitness status [51]. Furthermore, case–control studies have shown lower levels of PA in patients with IBS, while other researchers have found no significant association between PA and IBS. In this context, Omagari et al. [51] reported a high level of PA among patients with IBS compared to those without IBS.

Interestingly, our study on IBS patients from Southern Italy did not find a substantial difference in PA levels at baseline between patients and No IBS subjects. This was probably related to the fact that the IBS group consisted mainly of patients with mild and moderate IBS, according to the classification based on the IBS-SSS questionnaire. However, after evaluation through field tests, we observed lower PC in No IBS subjects compared to those with IBS, in agreement with previously reported findings [20].

Further studies are surely needed to clarify this link. Our results again support the recommendation to increase PA in subjects with IBS and confirm previous findings indicating a protective effect of PA on GI symptoms [20,52].

Although PA should be recommended for patients with IBS, as suggested in the literature [22,53], limited studies provide precise guidance on the exercise program with specific FITT principles (frequency, intensity, time, and type).

The current findings highlight the importance of objectively monitoring participants’ physical capabilities to implement effective interventions. A three-month moderate-intensity walking regimen, performed thrice weekly, yielded several advantages, especially for IBS patients. As measured by GPCS, augmented PC resulted in a significant reduction in IBS symptoms in this cohort. The study indicates that achieving an “above the average” score is crucial for eliciting statistically significant outcomes, making GPCS a valuable prognostic tool for personalized treatment criteria.

Interestingly, the adherence of the walking group to the program was total in our study, attributed to the enjoyment participants experienced during the exercise [54]. Indeed, it is well known that walking groups are successful both in contributing to the improvement of participants’ health and well-being and in attracting a large number of people at the same time, with low levels of drop-out [55]. Furthermore, the benefits of this type of training were evidenced by the participant’s willingness to continue walking even after the project was completed, so the exercise intervention was not just an end but the start of a change in the participants’ lifestyle.

Some methodological issues need to be considered. The program’s main strength was its supervised nature by trained personnel. Despite the relatively small number of participants per group, three trainers were assigned to each session to establish a personalized connection with each participant, resulting in full program adherence. The training protocol was meticulously designed, adhering to the FITT principles’ specific parameters and guidelines established by major international associations [56]. The exercise prescription was provided from a dose–response perspective to achieve the best results for individuals with IBS. Moreover, the presented results possess practical clinical applications and do not necessitate costly resources.

Further studies could be useful for advancing our knowledge about the relationship between physical exercise and IBS by exploring a broader range of activities and refining the prescription process for better, more personalized outcomes. This approach could have the potential to contribute significantly to the development of effective and individualized interventions for managing IBS symptoms through PA.

Nevertheless, certain limitations should be acknowledged. Although the GPCS has been utilized in prior studies, it needs formal validation. However, this score relies on three tests widely recognized in the literature for measuring physical capabilities. These tests are validated, repeatable, reproducible, and objective. Additionally, the small sample size may only represent a subset of the IBS population. Nevertheless, similar studies have shown that a comparable sample size was sufficient to detect significant differences in PA exposure.

## 5. Conclusions

Moderate-intensity aerobic exercise, performed for at least 180 min per week, can improve GI symptoms in patients with IBS and should, therefore, be added to the list of recommended primary interventions for patients with IBS. Additionally, monitoring the GPCS of IBS patients offers valuable insights into the correlation between PA and symptom severity. Through continuous PC monitoring, healthcare professionals can evaluate the impact of exercise and lifestyle modifications on the overall well-being of these patients. This assessment may reveal improvements in bowel habits, reduced pain levels, and an enhanced quality of life. Furthermore, regular monitoring allows healthcare professionals to tailor treatment plans and interventions more effectively, addressing specific symptoms and optimizing outcomes for patients with IBS.

## Figures and Tables

**Table 1:** Characteristics of participants.

		Exercise Intervention		
	No IBS (n.38)	IBS Pre (n. 40)	IBS Post (40)	*p*
Sex (male/female)	21M/17F	11M/29F		
Age (years)	53.71 ± 7.27	52.10 ± 7.72		
Body mass index	29.96 ± 5.84 ^a^	29.04 ± 5.12 ^a^	28.80 ± 5.15 ^a^	0.0690
Waist circumference	98.69 ± 13.71 ^a^	93.04 ± 13.41^a^	92.71 ± 13.62 ^a^	0.1149
Hip circumference	106.53 ± 11.26 ^a^	106.22 ± 10.07 ^a^	105.51 ± 10.07 ^a^	0.0465
Waist/hip ratio	0.93 ± 0.11 ^a^	0.87 ± 0.12 ^a^	0.88 ± 0.12 ^a^	0.0899
PhA (degrees)	6.76 ± 1.06 ^a^	6.46 ± 1.07 ^a^	6.44 ± 0.98 ^a^	0.3671
BCM (kg)	32.51 ± 7.22 ^a^	28.90 ± 7.04 ^b^	28.62 ± 6.6 ^b^	0.0380
FM (kg)	27.82 ± 12.10 ^a^	27.21 ± 10.84 ^a^	26.72 ± 10.91 ^a^	0.9734
FFM (kg)	56.70 ± 10.14 ^a^	51.60 ± 9.29 ^b^	51.22 ± 8.88 ^b^	0.0292
TBW (liters)	41.32 ± 7.68 ^a^	37.60 ± 6.84 ^b^	37.33 ± 6.44 ^b^	0.0340
ECW (liters)	17.57 ± 3.35 ^a^	16.44 ± 2.64 ^a^	16.36 ± 2.51 ^a^	0.2289
Global Physical Capacity Score	2.21 ± 1.76 ^a^	2.40 ± 1.53 ^a^	3.32 ± 1.68 ^b^	<0.0001
IBS scores				
Abdominal pain intensity	1.84 ± 6.62 ^a^	24.50 ± 27.26 ^b^	12.58 ± 21.92 ^b^	<0.0001
Abdominal pain frequency	1.58 ± 5.94 ^a^	21.75 ± 29.86 ^b^	8.62 ± 18.15 ^b^	0.0003
Abdominal Distension	9.21 ± 14.36 ^a^	45.75 ± 23.95 ^b^	27.80 ± 21.37 ^c^	<0.0001
Dissatisfaction with bowel habits	10.39 ± 12.81^a^	47.75 ± 32.20 ^b^	31.25 ± 23.88 ^b^	0.0002
Interference on life in general	5.39 ± 9.96 ^a^	43.38 ± 25.68 ^b^	31.50 ± 27.20 ^b^	0.0077
Total score	28.45 ± 26.02 ^a^	183.10 ± 79.43 ^b^	111.80 ± 76.84 ^c^	<0.0001
2Km Walking Test				*p* *
Under the mean	20 (52.6%)	21 (52.5%)	11 (27.5%)	
In mean	16 (42.1%)	15 (37.5%)	20 (50%)	0.0516
Above the mean	2 (5.3%)	4 (10.0%)	9 (22.5%)	
Sit and Reach Test				
Under the mean	23 (60.5%)	22 (55.0%)	16 (40%)	
In mean	2 (5.3%)	4 (10.0%)	4 (10%)	0.4907
Above the mean	13 (34.2%)	14 (35.0%)	20 (50%)	
Hand-Grip Test				
Under the mean	14 (36.8%)	12 (30.0%)	9 (22.5%)	
In mean	12 (31.6%)	15 (37.5%)	11 (27.5%)	0.3771
Above the mean	12 (31.6%)	13 (32.5%)	20 (50%)	
IPAQ Categories °				
<700	13 (34.2%)	11 (27.5%)		
700–2519	19 (50.0%)	19 (47.5%)		0.5723
≥2520	6 (15.8%)	10 (25.0%)		

BMI: body mass index; PhA: phase angle; BCM: body cell mass; FM: fat mass; FFM: fat-free mass; TBW: total body water; ECW: extracellular water. IPAQ: International Physical Activity Questionnaire. Continuous data reported as mean ± SD. Categorical data represented as numbers and percentages. At the Kruskal–Wallis with Dunn’s post-test, different letters differ significantly. *p*: significance obtained by Wilcoxon rank sum test. *p* *: Significance obtained with the chi-square test. °: IPAQ categories expressed in MET (metabolic equivalent of task). Different superscript letters differ significantly at the Kruskal–Wallis, with Dunn’s post-test used.

**Table 2:** Multilevel mixed-effects ordered logistic regression: effect of physical capacity on irritable bowel syndrome (IBS) severity.

GPCS	Odds Ratio	*p*-Value	95% CI
Below the mean	1.00		
In mean	0.32	0.10	[0.08, 1.27]
Above the mean	0.04	0.00	[0.00, 0.31]

Adjusted for age, sex, BMI, waist and hip circumference, and IBS categories. GPCS: Global Physical Capacity Score; var (subjects): estimated variance of subjects. var (subjects) 8.28 [3.37, 20.34].

## Data Availability

Data are available upon reasonable request.

## Appendix A

**Table A1:** Temporal trend of GPCS and IBS-SSS scores and some anthropometrical data during intervention time.

	No IBS	
	Exercise Intervention	
	**PRE**	**POST**
N.	21M/17F	21M/17F
GPCS	2.21 ± 1.76	3.13 ± 1.88
IBS-SSS	28.45 ± 26.02	22.26 ± 28.78
BMI	29.96 ± 5.84	29.47 ± 5.45
WC	98.69 ± 13.71	96.32 ± 11.19
HC	106.53 ± 11.26	106.27 ± 10.02

N: number of subjects; GPCS: Global Physical Capacity Score; IBS-SSS: IBS Severity Scoring System; BMI: Body Mass Index; WC: Waist Circumference; HC: Hip Circumference. Data are reported as mean ± SD.

## References

[1] Vandvik P.O., Lydersen S., Farup P.G. (2006). Prevalence, comorbidity and impact of irritable bowel syndrome in Norway. Scand. J. Gastroenterol..

[2] Wilson S., Roberts L., Roalfe A., Bridge P., Singh S. (2004). Prevalence of irritable bowel syndrome: A community survey. Br. J. Gen. Pract..

[3] Thompson W.G., Longstreth G., Drossman D., Heaton K., Irvine E., Müller-Lissner S. (1999). Functional bowel disorders and functional abdominal pain. Gut.

[4] Kearney D.J., Brown-Chang J. (2008). Complementary and alternative medicine for IBS in adults: Mind–body interventions. Nat. Clin. Pract. Gastroenterol. Hepatol..

[5] Spanier J.A., Howden C.W., Jones M.P. (2003). A systematic review of alternative therapies in the irritable bowel syndrome. Arch. Intern. Med..

[6] Wald A., Rakel D. (2008). Behavioral and complementary approaches for the treatment of irritable bowel syndrome. Nutr. Clin. Pract..

[7] Wu J.C. (2010). Complementary and alternative medicine modalities for the treatment of irritable bowel syndrome: Facts or myths?. Gastroenterol. Hepatol..

[8] Cozma-Petruţ A., Loghin F., Miere D., Dumitraşcu D.L. (2017). Diet in irritable bowel syndrome: What to recommend, not what to forbid to patients!. World J. Gastroenterol..

[9] Ooi S.L., Correa D., Pak S.C. (2019). Probiotics, prebiotics, and low FODMAP diet for irritable bowel syndrome–What is the current evidence?. Complement. Ther. Med..

[10] Orlando A., Tutino V., Notarnicola M., Riezzo G., Linsalata M., Clemente C., Prospero L., Martulli M., D’Attoma B., De Nunzio V. (2020). Improved symptom profiles and minimal inflammation in IBS-D patients undergoing a long-term low-FODMAP diet: A lipidomic perspective. Nutrients.

[11] Riezzo G., Prospero L., Orlando A., Linsalata M., D’Attoma B., Ignazzi A., Giannelli G., Russo F. (2023). A Tritordeum-Based Diet for Female Patients with Diarrhea-Predominant Irritable Bowel Syndrome: Effects on Abdominal Bloating and Psychological Symptoms. Nutrients.

[12] Blumenthal J.A., Babyak M.A., Doraiswamy P.M., Watkins L., Hoffman B.M., Barbour K.A., Herman S., Craighead W.E., Brosse A.L., Waugh R. (2007). Exercise and pharmacotherapy in the treatment of major depressive disorder. Psychosom. Med..

[13] Mannerkorpi K. (2005). Exercise in fibromyalgia. Curr. Opin. Rheumatol..

[14] Steindorf K., Jedrychowski W., Schmidt M., Popiela T., Penar A., Galas A., Wahrendorf J. (2005). Case-control study of lifetime occupational and recreational physical activity and risks of colon and rectal cancer. Eur. J. Cancer Prev..

[15] Wolin K.Y., Lee I.M., Colditz G.A., Glynn R.J., Fuchs C., Giovannucci E. (2007). Leisure-time physical activity patterns and risk of colon cancer in women. Int. J. Cancer.

[16] Zhou C., Zhao E., Li Y., Jia Y., Li F. (2019). Exercise therapy of patients with irritable bowel syndrome: A systematic review of randomized controlled trials. Neurogastroenterol. Motil..

[17] Costantino A., Pessarelli T., Vecchiato M., Vecchi M., Basilisco G., Ermolao A. (2022). A practical guide to the proper prescription of physical activity in patients with irritable bowel syndrome. Dig. Liver Dis..

[18] Belvederi Murri M., Folesani F., Zerbinati L., Nanni M.G., Ounalli H., Caruso R., Grassi L. (2020). Physical activity promotes health and reduces cardiovascular mortality in depressed populations: A literature overview. Int. J. Environ. Res. Public Health.

[19] Villoria A., Serra J., Azpiroz F., Malagelada J.-R. (2006). Physical activity and intestinal gas clearance in patients with bloating. Off. J. Am. Coll. Gastroenterol. ACG.

[20] Dainese R., Serra J., Azpiroz F., Malagelada J.-R. (2004). Effects of physical activity on intestinal gas transit and evacuation in healthy subjects. Am. J. Med..

[21] Johannesson E., Ringström G., Abrahamsson H., Sadik R. (2015). Intervention to increase physical activity in irritable bowel syndrome shows long-term positive effects. World J. Gastroenterol. WJG.

[22] Johannesson E., Simrén M., Strid H., Bajor A., Sadik R. (2011). Physical activity improves symptoms in irritable bowel syndrome: A randomized controlled trial. Off. J. Am. Coll. Gastroenterol. ACG.

[23] Physical Activity Guidelines Advisory Committee (2008). Physical Activity Guidelines Advisory Committee Report.

[24] Lee I.-M., Skerrett P.J. (2001). Physical activity and all-cause mortality: What is the dose-response relation?. Med. Sci. Sports Exerc..

[25] Murphy M.H., Nevill A.M., Murtagh E.M., Holder R.L. (2007). The effect of walking on fitness, fatness and resting blood pressure: A meta-analysis of randomised, controlled trials. Prev. Med..

[26] Hu F.B., Sigal R.J., Rich-Edwards J.W., Colditz G.A., Solomon C.G., Willett W.C., Speizer F.E., Manson J.E. (1999). Walking compared with vigorous physical activity and risk of type 2 diabetes in women: A prospective study. JAMA.

[27] Fox K. (2004). At Least Five a Week: Evidence on the Impact of Physical Activity and Its Relationship to Health—A Report from the Chief Medical Officer.

[28] MIND (2008). The MIND Guide to Physical Activity.

[29] Kwak L., Kremers S., Walsh A., Brug H. (2006). How is your walking group running?. Health Educ..

[30] Doughty K. (2013). Walking together: The embodied and mobile production of a therapeutic landscape. Health Place.

[31] Pate R.R. (1983). A new definition of youth fitness. Physician Sportsmed..

[32] Caspersen C.J., Powell K.E., Christenson G.M. (1985). Physical activity, exercise, and physical fitness: Definitions and distinctions for health-related research. Public Health Rep..

[33] Lee P.H., Macfarlane D.J., Lam T.H., Stewart S.M. (2011). Validity of the international physical activity questionnaire short form (IPAQ-SF): A systematic review. Int. J. Behav. Nutr. Phys. Act..

[34] Khalil S.F., Mohktar M.S., Ibrahim F. (2014). The theory and fundamentals of bioimpedance analysis in clinical status monitoring and diagnosis of diseases. Sensors.

[35] Laukkanen R., Oja P., Pasanen M., Vuori I. (1992). Validity of a two kilometre walking test for estimating maximal aerobic power in overweight adults. Int. J. Obes. Relat. Metab. Disord. J. Int. Assoc. Study Obes..

[36] Pearn J., Bullock K. (1979). A portable hand-grip dynamometer. J. Paediatr. Child Health.

[37] Hoeger W.W., Hopkins D.R. (1992). A comparison of the sit and reach and the modified sit and reach in the measurement of flexibility in women. Res. Q. Exerc. Sport.

[38] Tanaka H., Monahan K.D., Seals D.R. (2001). Age-predicted maximal heart rate revisited. J. Am. Coll. Cardiol..

[39] Foster C., Porcari J.P., Anderson J., Paulson M., Smaczny D., Webber H., Doberstein S.T., Udermann B. (2008). The talk test as a marker of exercise training intensity. J. Cardiopulm. Rehabil. Prev..

[40] Wilson R.C., Jones P. (1989). A comparison of the visual analogue scale and modified Borg scale for the measurement of dyspnoea during exercise. Clin. Sci..

[41] Bouchard D.R., Soucy L., Sénéchal M., Dionne I.J., Brochu M. (2009). Impact of resistance training with or without caloric restriction on physical capacity in obese older women. Menopause.

[42] Francis C.Y., Morris J., Whorwell P.J. (1997). The irritable bowel severity scoring system: A simple method of monitoring irritable bowel syndrome and its progress. Aliment. Pharmacol. Ther..

[43] Warburton D.E., Bredin S.S. (2017). Health benefits of physical activity: A systematic review of current systematic reviews. Curr. Opin. Cardiol..

[44] Warburton D.E., Charlesworth S., Ivey A., Nettlefold L., Bredin S.S. (2010). A systematic review of the evidence for Canada’s Physical Activity Guidelines for Adults. Int. J. Behav. Nutr. Phys. Act..

[45] Costa R., Snipe R., Kitic C., Gibson P. (2017). Systematic review: Exercise-induced gastrointestinal syndrome—Implications for health and intestinal disease. Aliment. Pharmacol. Ther..

[46] Peters H.P., De Vries W.R., Vanberge-Henegouwen G.P., Akkermans L.M. (2001). Potential benefits and hazards of physical activity and exercise on the gastrointestinal tract. Gut.

[47] Monda V., Villano I., Messina A., Valenzano A., Esposito T., Moscatelli F., Viggiano A., Cibelli G., Chieffi S., Monda M. (2017). Exercise Modifies the Gut Microbiota with Positive Health Effects. Oxidative Med. Cell. Longev..

[48] Madsbad S. (2014). The role of glucagon-like peptide-1 impairment in obesity and potential therapeutic implications. Diabetes Obes. Metab..

[49] Nieman D.C., Wentz L.M. (2019). The compelling link between physical activity and the body’s defense system. J. Sport Health Sci..

[50] Caes L., Orchard A., Christie D. (2017). Connecting the mind–body split: Understanding the relationship between symptoms and emotional well-being in chronic pain and functional gastrointestinal disorders. Healthcare.

[51] Omagari K., Murayama T., Tanaka Y., Yoshikawa C., Inoue S.-i., Ichimura M., Hatanaka M., Saimei M., Muto K., Tobina T. (2013). Mental, physical, dietary, and nutritional effects on irritable bowel syndrome in young Japanese women. Intern. Med..

[52] De Schryver A.M., Keulemans Y.C., Peters H.P., Akkermans L.M., Smout A.J., De Vries W.R., Van Berge-Henegouwen G.P. (2005). Effects of regular physical activity on defecation pattern in middle-aged patients complaining of chronic constipation. Scand. J. Gastroenterol..

[53] Colwell L., Prather C., Phillips S., Zinsmeister A. (1998). Effects of an irritable bowel syndrome educational class on health-promoting behaviors and symptoms. Am. J. Gastroenterol..

[54] Parfitt G., Hughes S. (2009). The exercise intensity–affect relationship: Evidence and implications for exercise behavior. J. Exerc. Sci. Fit..

[55] Hanson S., Jones A. (2015). Is there evidence that walking groups have health benefits? A systematic review and meta-analysis. Br. J. Sports Med..

[56] Thompson P.D., Arena R., Riebe D., Pescatello L.S. (2013). ACSM’s new preparticipation health screening recommendations from ACSM’s guidelines for exercise testing and prescription. Curr. Sports Med. Rep..

